# Probiotic *Pediococcus pentosaceus* Li05 Improves Cholestasis through the FXR-SHP and FXR-FGF15 Pathways

**DOI:** 10.3390/nu15234864

**Published:** 2023-11-22

**Authors:** Shengyi Han, Kaicen Wang, Jian Shen, He Xia, Yanmeng Lu, Aoxiang Zhuge, Shengjie Li, Bo Qiu, Shuobo Zhang, Xiangmin Dong, Mingfei Yao, Lanjuan Li

**Affiliations:** 1 State Key Laboratory for Diagnosis and Treatment of Infectious Diseases, National Clinical Research Center for Infectious Diseases, National Medical Center for Infectious Diseases, Collaborative Innovation Center for Diagnosis and Treatment of Infectious Diseases, The First Affiliated Hospital, Zhejiang University School of Medicine, 79 Qingchun Rd., Hangzhou 310003, China; 2 Sir Run Run Shaw Hospital, Zhejiang University School of Medicine, Hangzhou 310000, China; 3 Jinan Microecological Biomedicine Shandong Laboratory, Jinan 250000, China

**Keywords:** cholestasis, primary sclerosing cholangitis, *Pediococcus pentosaceus*, gut microbiota, bile acid, farnesoid X receptor

## Abstract

Primary sclerosing cholangitis (PSC), a rare chronic cholestatic liver disease, is characterized by intrahepatic or extrahepatic strictures accompanied by biliary fibrosis. So far, there are no effective therapies to slow down the progression of this disease. Farnesoid X receptors (FXRs) are ligand-activated transcription factors involved in the control of bile acid (BA) synthesis and enterohepatic circulation. Therefore, targeting FXRs holds promise as a potential approach for treating PSC. *Pediococcus pentosaceus* Li05 is a probiotic that was isolated from healthy volunteers and has previously been shown to have an anti-inflammatory effect in DSS-induced colitis. In this study, we established a 3,5-diethoxycarbonyl-1,4-Dihydrocollidine (DDC)-induced cholestasis mouse model and investigated the effects of *Pediococcus pentosaceus* Li05 on PSC. Our findings revealed that administration of Li05 significantly attenuated liver damage, hepatic inflammation, and fibrosis, as well as bile duct hyperplasia. Li05 activated the hepatic FXR-SHP and ileal FXR-FGF15 signaling pathways to decrease the expression of Cyp7a1. In addition, the Li05-modulated gut microbiota structure especially improved the abundance of 7α-dehydroxylation bacteria like *Eubacterium*. The intervention of Li05 also improved the intestinal barrier and reduced bacterial endotoxin translocation. Based on these findings, Li05 shows promise for future application as a therapeutic strategy for cholestasis.

## 1. Introduction

Primary sclerosing cholangitis (PSC) is a rare disease characterized by multifocal bile duct strictures and progressive liver disease [1]. The development of bile duct stenosis in PSC can be attributed to chronic hepatic inflammation and fibrosis [2]. Meanwhile, bile duct stenosis causes an accumulation of bile acids (BAs) in the liver, and cytotoxic BAs further result in hepatic fibrosis, cirrhosis, and even hepatocellular carcinoma [3].

The farnesoid X receptor (FXR), a BA-activated transcription factor, plays a critical role in BA homeostasis [4]. The FXR is mainly distributed in the liver, ileum, and kidney [5]. Activation of the FXR by BAs upregulates small heterodimer partner (SHP) in the liver, fibroblast growth factor 15 (FGF15) in the ileum, and BA transporters to regulate BA synthesis, degradation, secretion, and absorption [4]. Obeticholic acid (OCA), an approved clinical agent for PSC, activates the FXR to decrease bile synthesis and increase bile excretion [6]. However, to date, there are no effective drugs that can delay disease progression or improve survival in transplant-free patients [7]. Therefore, it remains imperative to explore novel clinical treatments for PSC.

Probiotics are live microorganisms that can provide health benefits to the host when given in sufficient amounts [8]. The administration of probiotics alters the intestinal microecological composition and participates in metabolic synthesis, including short-chain fatty acids (SCFAs) [9], tryptophan catabolites [10], and BAs [11]. The bidirectional relationship between the gut and liver facilitates the direct transportation of gut-derived products to the liver [12]. Numerous studies have demonstrated the therapeutic potential of various probiotics in animal models for liver diseases, including acute liver injury [13], non-alcoholic fatty liver disease [14], cirrhosis [15], and PSC [16,17]. A probiotic strain named *Pediococcus pentosaceus* Li05 was isolated from healthy volunteers in our laboratory [18]. Our previous work demonstrated that the administration of Li05 can confer protection against colitis by enhancing the intestinal barrier [19] and enhance survival in mice with liver cirrhosis by attenuating hepatic inflammation [20]. However, the efficacy of Li05 in alleviating the symptoms of PSC and the underlying mechanism remain elusive. In this study, we used a diet containing 0.1% 3,5-diethoxycarbonyl-1,4-dihydrocollidine (DDC) to induce PSC. DDC promotes the accumulation of protoporphyrin, blocks small bile ducts, further damages the bile duct epithelium, and causes liver injury along with ductal reactions [21]. Our objective is to assess the protective effect of Li05 against PSC and elucidate potential molecular mechanisms, providing novel evidence for the treatment of cholestatic liver disease.

## 2. Materials and Methods

### 2.1. Strain and Culture Conditions

*Pediococcus pentosaceus* Li05 (CGMCC 7049) was cultured in Man Rogosa Sharpe (MRS) medium (Oxoid, Basingstoke, UK) under anaerobic conditions for one day. Subsequently, Li05 was harvested by centrifugation (5000× *g*, 10 min). After washing twice using sterile phosphate-buffered saline (PBS), Li05 was resuspended in an appropriate volume of PBS to a concentration of 1 × 10^9^ CFU (colony-forming units)/mL [19].

### 2.2. Set-Up of the Mouse Model and Experimental PSC Induction

**Figure 1:**
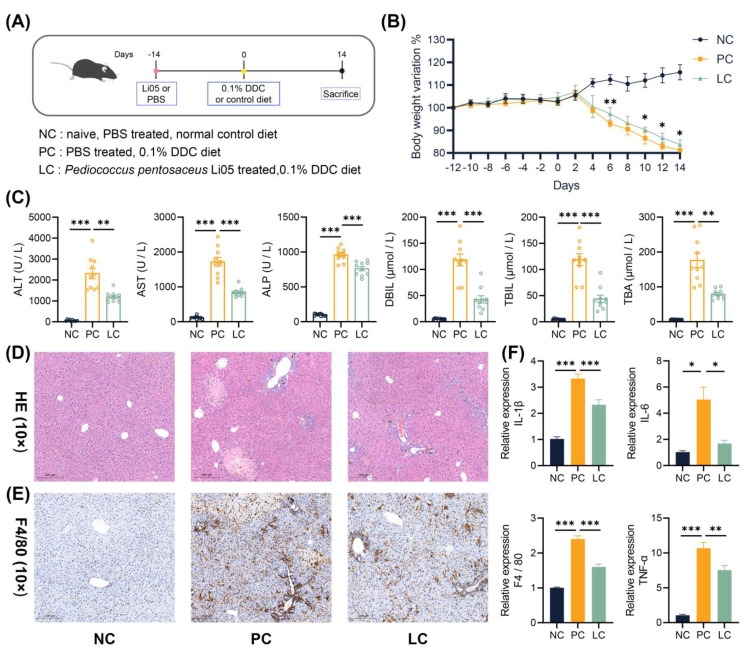
Li05 improves liver damage and inflammation. (**A**) Schematic diagram of the mouse experiment. (**B**) Body weight variation in mice recorded from day –12 to day 14 of the experiment in the NC, PC, and LC groups. (**C**) Liver functions (ALT, AST, ALP, TB, DB, and TBA) of the serum. (**D**) Representative images of the H&E staining of liver tissue. (**E**) Representative images of F4/80 immunohistochemistry-stained preparations of the liver tissue. (**F**) Hepatic IL-1β, IL-6, TNF-α, and F4/80 expressions were determined by qRT-PCR. Data are presented as mean ± SEM, * *p* < 0.05, ** *p* < 0.01, and *** *p* < 0.001. *p* values were calculated using a one-way ANOVA test.

Specific pathogen-free (SPF) male, eight-week-old C57/BL6J mice were purchased from Ziyuan Experimental Animal Technology (Hangzhou, China) and housed under controlled 12 h light and dark cycles. Mice were randomly assigned to three groups: normal control (NC), PBS + DDC diet (PC), and Li05 + DDC diet (LC) groups (n = 10 in each group). Mice in the LC group received 200 μL of P. pentosaceus Li05 solution by oral gavage daily from day –14 to day 14. Accordingly, mice in the NC and PC groups were treated with 200 μL of PBS by oral gavage daily as controls (Figure 1A). Starting from day 0, mice in the PC and LC groups were fed a 0.1% DDC diet (D17142193, Future Biotech, Beijing, China), and mice in the NC group were fed a control diet (D13112201, Future Biotech, Beijing, China). The healthy conditions (including movement, fur color, and stool trait) of mice were monitored every day, and weight was recorded every other day. On day 14, all mice were sacrificed.

### 2.3. Histopathology, Immunohistochemistry, and Immunofluorescence Analysis

A total of 0.5 cm × 0.5cm × 0.5 cm of liver and 1 cm of proximal colon tissues were fixed in 4% paraformaldehyde overnight at RT. Then tissues were embedded in paraffin and cut into 3 μm-thick sections. Following deparaffinization and hydration, the tissue sections were stained with a hematoxylin and eosin solution. The samples were observed under a light microscope. For immunohistochemistry, liver sections were stained with Picro Sirius Red Stain (PSR) (Solarbio, Beijing, China), F4/80, α-SMA, and CK-19 (Abcam, Cambridge, UK). The positive area of PSR, F4/80, α-SMA, and CK-19 staining was assessed by the ImageJ IHC profiler. For immunofluorescence, colon sections were stained with occludin (Abcam, Cambridge, UK).

### 2.4. Transmission Electron Microscopy (TEM)

Colon tissues were collected and fixed immediately in a 2.5% glutaraldehyde solution in a dark environment (24 h, 4 °C). The colon was washed 3 times with PBS to remove glutaraldehyde. Then the tissues were fixed using 1% osmic acid and washed with PBS again. After complete dehydration, the samples were sectioned with an ultramicrotome (Leica, Wetzlar, Germany). Lead citrate solution and uranyl acetate solution were used to stain the samples successively. Finally, the prepared tissues were observed under a Tecnai G2 Spirit transmission electron microscope (Thermo Fisher Scientific, Waltham, MA, USA).

### 2.5. Serum Biochemical Tests and Cytokines, LBP Analysis, and Assay of TBA in the Liver

Serum liver function was measured by an automated biochemical analyzer (Hitachi 7600-210; Tokyo, Japan). Serum cytokines, including IL-1β, IL-2, IL-6, IL-10, INF-γ, MCP-1, and G-CSF, were assessed using a mouse inflammation array kit (FAM-INF-1, Raybiotech, Atlanta, GA, USA). Lipopolysaccharide-binding protein (LBP) was measured by a mouse LBP ELISA Kit (Abcam, Cambridge, UK). The TBA in the liver was detected by a TBA colorimetric assay kit (Elabscience, Wuhan, China).

### 2.6. RNA Extraction and Real-Time Quantitative PCR Analysis

The RNeasy kit (Qiagen, Hilden, Germany) was used for the extraction of total RNA from liver, ileum, and colon tissues. Then RNA was reverse transcribed to cDNA with PrimeScript RT master mix (TaKaRa Biomedicals, Kusatsu, Japan) and finally stored at −80 °C. The relative expression of targeted genes was analyzed in duplicate, and β-actin was used as an internal reference. Finally, ΔΔCT was calculated for the final measurement. Primers are presented in Table S1.

### 2.7. Fecal Bile Acid Composition Measurement

A total of 20 mg of fecal sample was accurately weighed and supplemented with 100 µL of 50% methanol. After homogenizing, an additional 500 µL of ice-cold acetonitrile (containing 5% ammonia) was added, and the extract was ultrasonicated in an ice water bath for 20 min. Subsequently, centrifugation was carried out at 18,000× *g* for 10 min at 4 °C, and a volume of 300 µL of the supernatant was taken to be analyzed using ultra-performance liquid chromatography-mass spectrometry (UPLC-ESI-MS, Agilent, Santa Clara, CA, USA).

### 2.8. Fecal SCFA Composition Measurement

The fecal sample (50 mg) was homogenized and centrifuged at 4 °C for 10 min at 15,000× *g*. Then the supernatant was extracted with phosphoric acid, a 4-methylvaleric acid solution, and ether. After another round of centrifugation, the supernatant was extracted for testing. The GC analysis was performed on a trace 1310 gas chromatograph (Thermo Fisher Scientific, Waltham, MA, USA).

### 2.9. 16S rRNA Analysis

Total fecal bacterial DNA was extracted with the DNeasy PowerSoil Pro kit (Qiagen, Hilden, Germany). The V3-V4 region gene was amplified using universal bacterial primers (see Table S1). The PCR products were detected with electrophoresis and purified with magnetic beads. Then samples were sequenced with the NovaSeq 6000 platform (Illumina, San Diego, CA, USA). According to the QIIME 2 (2020.11) default parameters, the qualified raw data were quality filtered, noise reduced, spliced, and de-chimerized to obtain representative sequences and ASV abundance tables. Finally, all representative sequences were annotated and compared to the database used for subsequent analysis.

### 2.10. Statistical Analysis

The results are presented as means ± SEM. The D’Agostino and Pearson, Anderson–Darling, Kolmogorov–Smirnov, and Shapiro–Wilk tests were used to analyze the normality of the data. Statistically significant differences between the three groups were evaluated by a one-way ANOVA test. The Brown-Forsythe ANOVA test was applied to the groups that did not show homogeneity of variance. The Kruskal–Wallis test was applied when the data did not pass the normal distribution test. A *p* < 0.05 was considered statistically significant. GraphPad Prism (version 8) was used for the statistical analyses.

## 3. Results

### 3.1. Li05 Ameliorates Liver Damage and Reduces the Inflammatory Response Induced by DDC

Figure 1B showed that mice weight was remarkably lower in the DDC dietary intake group than in the normal group. The administration of Li05 obviously mitigated the weight loss on days 6, 10, 12, and 14. Compared to the NC group, levels of ALT, AST, ALP, TB, DB, and TBA in the serum were significantly increased in the PC group (Figure 1C). Treatment with Li05 resulted in a significant reduction in these five liver function indexes. Through macroscopic examination, the livers in the PC and LC groups exhibited a dark brown color indicative of cholestasis. Histologic examination of liver sections revealed less intrabiliary cholestasis, proliferation of the bile ducts with inflammatory infiltration, and less severe hepatocellular necrosis in mice treated with Li05 compared to those in the PC group (Figure 1D). Additionally, immunohistochemistry staining for F4/80 demonstrated a significant reduction in macrophage infiltration in the liver tissue of the LC group when compared to the PC group (Figure 1E and Figure S1). Correspondingly, significantly lower levels of F 4/80, IL-1β, IL-6, and TNF-α transcripts in the liver were found in the LC group (Figure 1F). 

**Figure 2:**
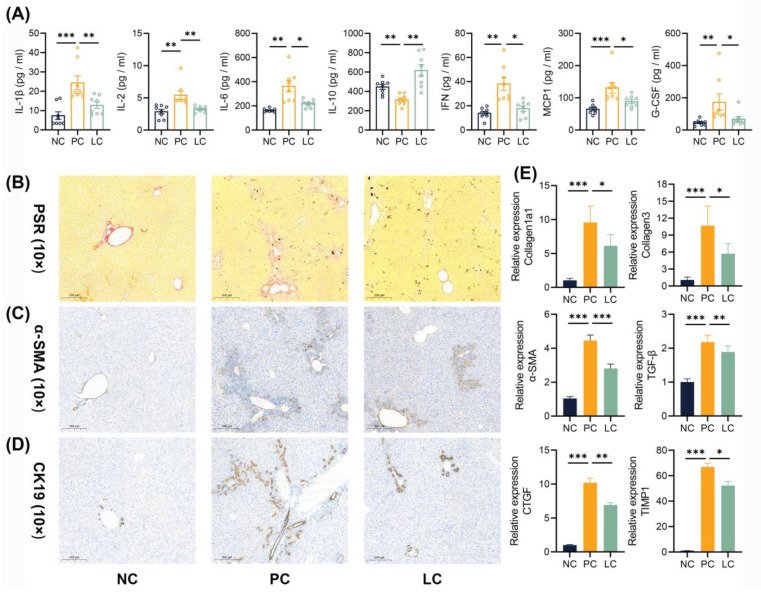
Li05 attenuates systemic inflammation, liver fibrosis, and bile duct hyperplasia. (**A**) Cytokines in the serum, including IL-1β, IL-2, IL-6, IL-10, IFN-γ, MCP1, and G-CSF. (**B**–**D**) Representative images of PSR, α-SMA, and CK-19 immunohistochemistry-stained preparations of the liver tissue. (**E**) Hepatic fibrosis genes, including Collagen1a1, Collagen3, α-SMA, TGF-β, CTGF, and TIMP1 expressions, were determined by qRT-PCR. Data are presented as mean ± SEM, * *p* < 0.05, ** *p* < 0.01, and *** *p* < 0.001. *p* values were calculated using a one-way ANOVA test or a Kruskal–Wallis test.

Aside from inflammation in the liver tissue, we detected increased levels of cytokines in the serum. Several pro-inflammatory cytokines, including IL-1β, IL-2, IL-6, INF-γ, MCP-1, and G-CSF, exhibited an obvious increase in the PC group compared to the NC group (Figure 2A). The treatment of Li05 reduced these pro-inflammatory cytokines while concurrently elevating anti-inflammatory IL-10 levels significantly.

### 3.2. Li05 Attenuates Liver Fibrosis and Bile Duct Hyperplasia Caused by DDC

The liver sections from the PC group exhibited severe fibrosis, as evidenced by intense staining for Picro Sirius Red (PSR) and α-SMA immunohistochemistry staining. Conversely, the LC group showed an obvious reduction in PSR and α-SMA staining intensity compared to the PC group. (Figure 2B,C and Figure S1) Correspondingly, the levels of Collagen1a1, Collagen3, α-SMA, TGF-β, CTGF, and TIMP1 transcripts in the liver were decreased in the LC group (Figure 2E). Liver CK-19 immunohistochemistry staining showed obvious bile duct proliferation in DDC dietary intake groups, and the treatment of Li05 attenuated this proliferative response effectively (Figure 2D and Figure S1).

### 3.3. Li05 Suppresses Bile Acid Synthesis in DDC-Induced PSC Mice

**Figure 3:**
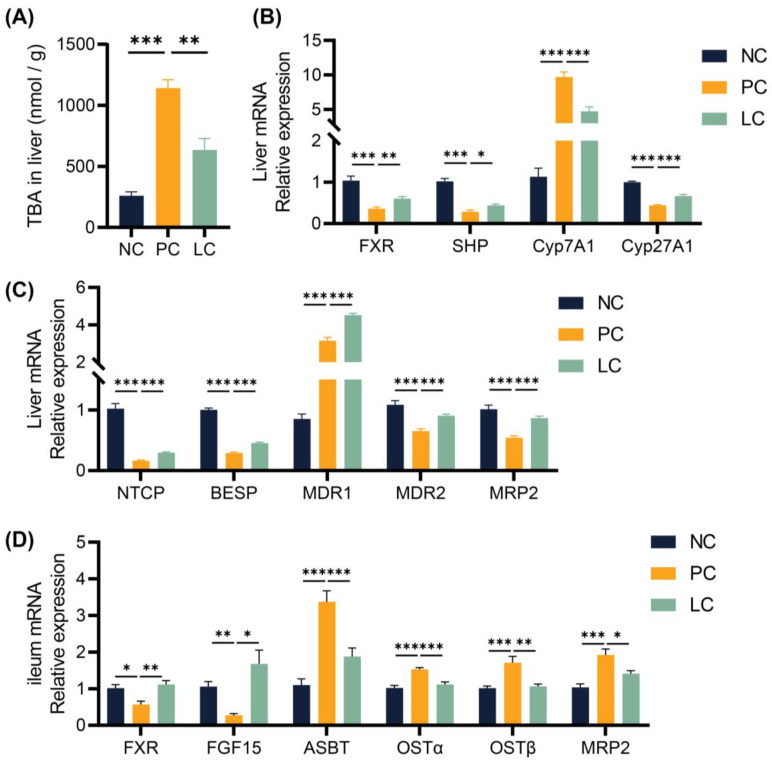
Li05 activated the FXR signaling pathway to regulate hepatic bile acid synthesis and transport. (**A**) The concentration of TBA in the liver. (**B**) Hepatic genes, including FXR, SHP, Cyp7a1, and Cyp27a1 expressions, were determined by qRT-PCR. (**C**) Hepatic BA transporter genes, including NTCP, BSEP, MDR1, MDR2, and MRP2 expressions, were determined by qRT-PCR. (**D**) FXR, FGF15, ASBT, OST α/β, and MRP2 expressions in the ileum were determined by qRT-PCR. Data are presented as mean ± SEM, * *p* < 0.05, ** *p* < 0.01, and *** *p* < 0.001. *p* values were calculated using a one-way ANOVA test.

To study the molecular mechanism of the protective effect of Li05 on DDC-induced PSC, we performed quantitative Real-time PCR (qRT-PCR) to examine the expression levels of the intrahepatic bile acid synthase, bile acid receptors, and hepatic and intestinal cyclic transfer proteins. Our findings revealed that the hepatic transcript of cholesterol 7α-hydroxylase (Cyp7a1) was upregulated and cholesterol 27-hydroxylase (Cyp27a1) was downregulated in the PC group compared to the NC group. However, treatment with Li05 resulted in a decrease in Cyp7a1 expression and a restoration of Cyp27a1 expression when compared to the PC group (Figure 3B). As a result, a decreased total BA level in the liver was found in the LC group (Figure 3A). Further results demonstrated that the expression of the FXR and SHP were significantly decreased in the PC group compared to the NC group, and the Li05 treatment restored the FXR and SHP expressions (Figure 3B). Meanwhile, the levels of Na+-taurocholate co-transporting polypeptide (NTCP), bile salt export pump (BSEP), multidrug resistance protein 2 (MDR2), and multidrug resistance-associated protein (MRP2) were inhibited in the PC group, while NTCP, BSEP, MDR2, and MRP2 were alleviated in the LC group. In contrast, the expression of multidrug resistance protein 1 (MDR1) was increased in the PC group, and it was even higher in the LC group (Figure 3C). 

In the ileum, the levels of the FXR and FGF15 were inhibited in the PC group. Compared to the PC group, Li05 treatment increased the FXR and FGF15 expressions (Figure 3D). In contrast, elevated levels of apical sodium-dependent bile acid transporter (ASBT), organic solute transporter subunit alpha (OSTα), OSTβ, and MRP2 were observed in the PC group, whereas administration of Li05 led to a reduction in these transport protein expressions (Figure 3D).

### 3.4. Li05 Relieves Hepatic Cholestasis by Increasing Fecal Excretion of BAs

**Figure 4:**
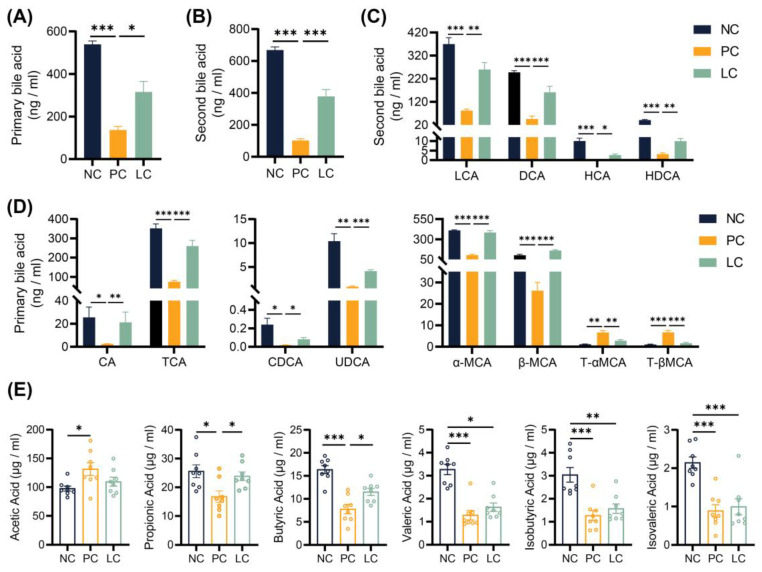
Alternation of Li05 on BA and SCFA metabolism. (**A**,**B**) The total primary and secondary bile acid concentration in feces. (**C**) Individual secondary bile acid concentration in feces. (**D**) Individual primary bile acid concentration in feces. (**E**) Six short-chain fatty acid concentrations in feces. Data are presented as mean ± SEM, * *p* < 0.05, ** *p* < 0.01, and *** *p* < 0.001. *p* values were calculated using a one-way ANOVA test or Kruskal–Wallis test.

In order to verify the difference in BAs excreted through feces, we quantified the concentration of BAs in fecal samples. The results revealed a significant decrease in concentrations of both primary and secondary BAs in the feces of mice in the PC group compared to the NC groups. Compared to the PC group, concentrations of total, primary, and secondary BAs in the LC group increased (Figure 4A,B). Specifically, the concentrations of six primary BAs, including CA, TCA, CDCA, UDCA, α-MCA, and β-MCA, decreased significantly in the PC group compared to the NC group. The concentrations of CA, TCA, CDCA, UDCA, α-MCA, and β-MCA were elevated in the LC group (Figure 4D). In contrast, concentrations of T-αMCA and T-βMCA were elevated in the PC group, while treatment with Li05 resulted in a decrease in both T-αMCA and T-βMCA levels (Figure 4D). Meanwhile, the concentrations of four primary BAs, including LCA, DCA, HCA, and HDCA, were significantly lower in the PC group compared to the NC group, and Li05 treatment increased LCA, DCA, HCA, and HDCA (Figure 4C).

### 3.5. Li05 Increases Intestinal Short-Chain Fatty Acid Concentration

Measurement of fecal SCFA concentrations revealed that the DDC diet increased the concentration of acetic acid while decreasing the concentration of propionic, butyric, isobutyric, valeric, and isovaleric acids when compared to the NC group (Figure 4E). The administration of Li05 elevated the concentration of propionic and butyric acids (Figure 4E). For isobutyric, valeric, and isovaleric acids, Li05 only caused an upward trend with no significant difference.

### 3.6. Effect of Li05 on Gut Microbiota Composition Alternation

**Figure 5:**
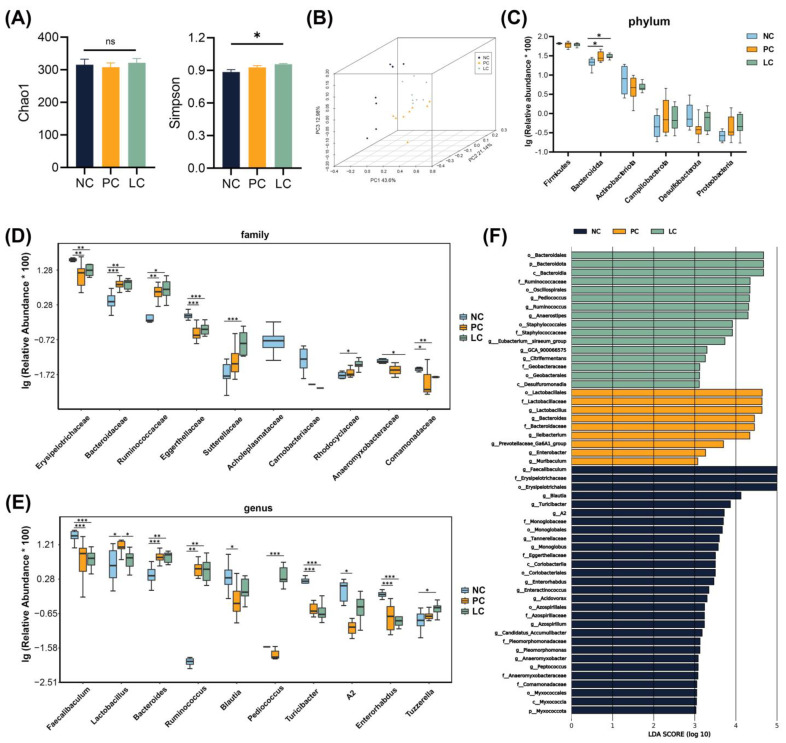
Analyses of the gut microbiota composition in feces samples. (**A**) Chao1 and Simpson indexes. (**B**) Weighted PCOA of the gut microbiota composition of mice in the three groups. (**C**–**E**) The top 10 taxa exhibiting significant differences at the phylum, family, and genus levels among the three groups. (**F**) LEfSe analyses in the three groups. Data are presented as mean ± SEM, * *p* < 0.05, ** *p* < 0.01, and *** *p* < 0.001. *p* values were calculated using a one-way ANOVA test or Kruskal–Wallis test.

We conducted 16S rRNA gene sequencing to explore the alternation of the gut microbiota composition. No significant differences were found between the NC and PC groups when assessing α diversity using the Chao1 and Simpson indexes. However, a slight increase in the Simpson index was noted in the LC group compared to the NC group (Figure 5A). Adonis analysis revealed a significant alternation in gut microbiota composition among the NC, PC, and LC groups (Figure 5B). 

At the phylum level (Figure 5C), compared to the NC group, the PC and LC groups were enriched with *Bacteroidota*. At the family level (Figure 5D), the relative abundance of *Bacteroidaceae* and *Ruminococcaceae* exhibited an increase, while *Erysipelotrichaceae*, *Eggerthellaceae*, and *Comamonadaceae* exhibited a decrease in the PC group compared to the NC group. The relative abundance of *Bacteroidaceae*, *Ruminococcaceae*, *Sutterellaceae*, and *Rhodocyclaceae* increased, whereas *Erysipelotrichaceae*, *Eggerthellaceae*, *Anaeromyxobacteraceae*, and *Comamonadaceae* decreased in the LC group compared to the NC group. At the genus level (Figure 5E), the relative abundance of *Lactobacillus*, *Bacteroides*, and *Ruminococcus* was elevated, while *Faecalibaculums*, *Blautia*, *Turicibacter*, and *Enterorhabdus* decreased in the PC group compared to the NC group. The relative abundance of *Bacteroides*, *Ruminococcus*, *Pediococcus*, and *Tuzzerella* was elevated, while *Faecalibaculums*, *Turicibacter,* and *Enterorhabdus* decreased in the LC group compared to the NC group. The relative abundance of *Lactobacillus* decreased significantly in the LC group compared to the PC group.

We conducted LEfSe analysis to further reveal prognostic microbial markers (Figure 5F). The microbiota structure of the PC group was reflected in the enrichment of *Lactobacillus*, *Bacteroides,* and *Enterobacter*. In contrast, *Pediococcus*, *Anaerostipes*, and *Eubacterium siraeum* were enriched in the LC group.

### 3.7. Li05 Restores the Intestinal Barrier

**Figure 6:**
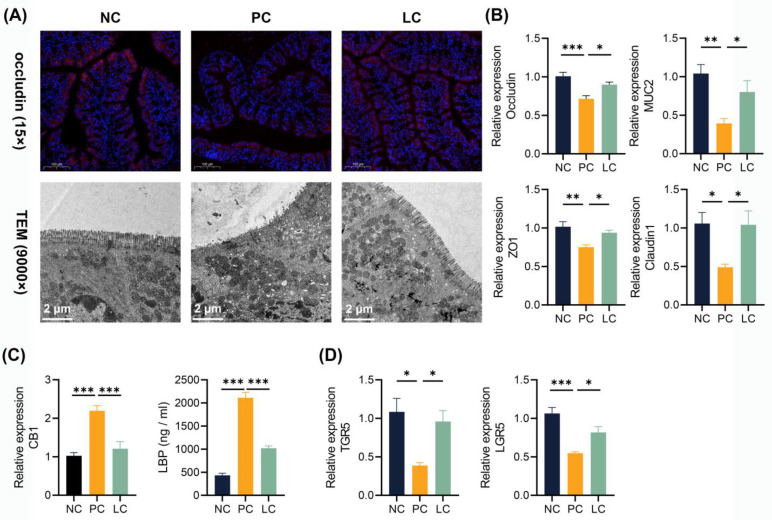
Li05 restored the intestinal barrier. (**A**) Representative images of occludin immunofluorescence-stained preparations and TEM images of the colon tissue. (**B**) Colonic occludin, ZO-1, MUC2, and claudin1 expressions were determined by qRT-PCR. (**C**) Colonic CB1 expression was determined by qRT-PCR and LBP levels in the serum. (**D**) Colonic TGR5 and LGR5 expressions were determined by qRT-PCR. Data are presented as mean ± SEM, * *p* < 0.05, ** *p* < 0.01, and *** *p* < 0.001. *p* values were calculated using a one-way ANOVA test.

To investigate the impact of the DDC diet on the intestinal barrier, we performed immunofluorescence staining of colon tissue and qRT-PCR to assess the expression levels of intestinal tight junction proteins. Colonic occludin immunofluorescence staining showed a decreased distribution of occludin in the PC group, and the intestinal barrier was more intact in the LC group compared to the PC group (Figure 6A). Meanwhile, the TEM results revealed that microvilli in the PC group exhibited ruptures and sparsity, while those in the LC group appeared denser and more intact (Figure 6A). Correspondingly, significantly lower levels of ZO-1, occludin, claudin1, and MUC2 levels of CB1 transcripts in the colon were found in the PC group. Compared to the PC group, the Li05 treatment restored the expression of ZO-1, occludin, claudin1, and MUC2 (Figure 6B). The level of cannabinoid G-coupled protein receptors (CB1) greatly increased in the PC group (Figure 6C). Consequently, a higher level of lipopolysaccharide-binding protein (LBP) was detected in the serum of the PC group, whereas the LBP level was significantly decreased in the LC group (Figure 6C). Furthermore, we observed the repression of both TGR5 and LGR5 transcript levels in the PC group. However, treatment with Li05 resulted in increased expression of TGR5 and LGR5 compared to the PC group (Figure 6D).

## 4. Discussion

In this study, we used a 0.1% DDC diet to induce cholestatic hepatitis to model PSC. The DDC diet induced symptoms of biliary fibrosis, hepatic cholestasis, hepatocellular necrosis, and a hepatic inflammatory response in mice by damaging bile duct epithelial cells [21]. Our findings demonstrated that liver function parameters such as ALT, AST, ALP, TB, DB, and TBA were considerably elevated in the PC group compared to the normal group, whereas these indexes were significantly decreased in the LC group. Additionally, Li05 treatment greatly reduced weight loss while also alleviating liver damage and decreasing cholestasis. 

Liver inflammation and fibrosis are characteristic of PSC [2]. Excessive deposition of BAs in the liver, due to bile duct strictures, leads to elevated pressure within the bile ducts and triggers inflammatory cell accumulation [7]. Meanwhile, BAs also activate intrahepatic inflammatory pathways, prompting macrophage accumulation and the production of cytokines such as IL-1β, IL-6, and TNF-α [22,23]. The persistence of inflammation, in turn, exacerbates liver injury and fibrosis [24]. Our previous study demonstrated that Li05 significantly inhibited the hepatic inflammatory response in liver cirrhosis [20]. Consistently, Li05 downregulated expression of intrahepatic IL-1β, IL-6, and TNF-α and reduced pro-inflammatory factors such as serum IL-1β, IL-2, IL-6, IFN-γ, and MCP1, preventing further liver injury due to inflammatory overload. The damage to bile duct epithelial cells induced by DDC stimulates inflammatory responses, and as a result, periportal liver fibrosis develops [25]. We observed a large decrease in the expression of several characteristic fibrosis genes [16,26], including Collagen1a1, Collagen3, α-SMA, TGF-β, CTGF, and TIMP1, in the LC group, demonstrating that the administration of Li05 effectively mitigated inflammation in the liver and suppressed the fibrotic process triggered by inflammation.

Negative feedback regulation of hepatic bile acid synthesis is conducted by BAs in two ways [27,28]. On the one hand, BAs can activate intrahepatic FXRs to upregulate SHP expression, thereby inhibiting Cyp7a1 expression [27]. On the other hand, BAs upregulate FGF15 through activation of ileal FXRs, and subsequently, FGF15 acts on intrahepatic FGFR4 through the portal vein, leading to downregulation of Cyp7a1 expression [27,29]. This hepatic-intestinal synergistic bile acid synthesis negative feedback mechanism plays a pivotal role in maintaining bile acid homeostasis [27]. Our findings demonstrated that supplementation with Li05 greatly upregulated the expression of the FXR and SHP in the liver as well as the FXR and FGF15 in the ileum. These results suggested that Li05 reduced cyp7a1 expression by activating the intrahepatic FXR-SHP and ileal FXR-FGF15 pathways. Further analysis revealed that FXR activation in the ileum may be attributed to the altered intestinal BA profile induced by the Li05 intervention. The administration of Li05 elevated the concentrations of CDCA, DCA, LCA, and CA with FXR-activating functions while decreasing the concentrations of T-αMCA and T-βMCA with FXR-inhibiting effects. It had been reported that T-βMCA was a naturally efficient FXR antagonist that hindered FXR activation with other BAs by occupying the binding site [30,31].

Normally, BAs synthesized by the liver are excreted through the bile ducts to the small intestine, with 90–95% being reabsorbed in the ileum and subsequently transported to the liver through the portal vein [32]. The bile acid transporter proteins are crucial for regulating BA circulation in the enterohepatic system. Among these transporters, BSEP serves as a vital efflux protein responsible for primary BA secretion from hepatocytes into bile canaliculi. In addition, MDR1/2 facilitates phosphatidylcholine transport, while MRP2 is involved in bilirubin glucuronide transport [27,28]. It has been reported that OSTα, OSTβ, MDR1/2, and MRP2 are under the transcriptional control of the FXR [28]. In our study, increased expressions of BSEP, MDR1, MDR2, and MRP2 were demonstrated in mice with Li05 treatment, leading to increased efflux of BAs from the liver and attenuation of bile accumulation. Moreover, the majority of unconjugated BAs are reabsorbed by the ASBT at the terminal ileum and subsequently secreted into the portal circulation through OSTα, OSTβ, and MRP2 [27,33]. Our results showed that the Li05 treatment decreased the levels of ASBT, OSTα, OSTβ, and MPR2 in the ileum and significantly increased the total BA concentration in the fecal samples, demonstrating that reabsorption of BAs in the ileum and influx of BAs in the portal vein were suppressed.

In conclusion, Li05 improved DDC-induced cholestasis in two ways. Firstly, the administration of Li05 enhanced the activation of the FXR in both the liver and ileum. In response, the levels of intrahepatic SHP and intestinal FGF15 were upregulated, collectively inhibiting Cyp7a1 expression and reducing BA synthesis at the source. Subsequently, enhanced hepatic and diminished intestinal expression of BA transporters affected by FXR activation together restored BA circulating homeostasis.

Bidirectional communication between the gut, its microbiota, and the liver is established through the portal vein [12]. Gut-derived products enter the liver through the portal vein, while BAs secreted by the liver regulate intestinal microecological composition [27]. The gut–liver axis provides a potential avenue for microecological therapy in treating liver diseases [13,16,20]. In our study, we used *Pediococcus pentosaceus* Li05 as a treatment and observed a significant increase in *Pediococcus* in the LC group, demonstrating a successful colonization of Li05 in the gut of mice. Meanwhile, we found the DDC diet induced a significant alternation in gut microbiota composition. A significant overrepresentation of *Lactobacillus* was found in PSC patients [34]. Consistently, our results described a higher relative abundance of *Lactobacillus* in the PC group, and the Li05 treatment decreased the level of *Lactobacillus*. *Anaerostipes* and *Eubacterium* were enriched in the LC group. *Anaerostipes* spp. metabolizes inositol to produce propionic acid and butyric acid [35]. *Eubacterium* spp. carries bile salt hydrolase (BSH) and converts unconjugated primary bile acids CDCA and CA to the secondary bile acids LCA and DCA by 7α-dehydroxylation [32,36]. Alterations of these microbiota also had an effect on BA pool composition and modulated the process of live damage.

Some liver diseases, including alcoholic liver disease [37] and cirrhosis [38], are associated with alterations in the intestinal microbiota and damage to the intestinal barrier. There is a close relationship between PSC and IBD [39]. About 50% to 80% of people diagnosed with PSC develop inflammatory bowel disease (IBD) [40]. Meanwhile, about 1–8% of IBD patients are diagnosed with PSC [39]. Impaired BA homeostasis in PSC patients leads to gut microbiota dysbiosis and disruption of the intestinal barrier [39]. Consistent with this, our results showed an expression decrease in colonic tight junction proteins, including ZO-1, occludin, and claudin1, in the DDC diet group. Meanwhile, as the regulator of gut permeability [41], CB1 showed an increase in the PC group. Due to the damage to the intestinal barrier and high intestinal permeability, bacteria and endotoxins migrate through the damaged intestines into the liver, activating the intrahepatic inflammasome and further exacerbating hepatic inflammation [42]. In contrast, the administration of Li05 increased the levels of ZO-1, occludin, and claudin and decreased the levels of CB1. The Li05 treatment protected intestinal barrier integrity, reduced bacterial translocation, and decreased the level of serum LBP. In DDC-induced PSC, significant alterations were observed in the bile acid metabolic profile. Primary and secondary BAs, especially LCA, DCA, CDCA, and CA, in the PC group decreased significantly. The expression of TGR5 and LGR5 in the colon was suppressed in the PC group. TGR5, a bile acid-specific G protein-coupled receptor, participates in various intracellular signaling pathways, including energy production, intestinal motility, and the inflammatory response [43]. Leucine-rich repeat-containing G protein-coupled receptor 5 (LGR5) is a marker gene of intestinal stem cells [44]. LCA, DCA, CDCA, and CA are effective activators of TGR5 [45]. Previous studies demonstrated that TGR5 activation had a protective effect on intestinal barrier integrity in DSS-induced colitis [46,47]. Giovanni Sorrentino et al. demonstrated that BAs promoted intestinal epithelial cell (IEC) regeneration by activating TGR5 in the ISC [48]. We speculate that the reduced concentration of bile acids, especially LCA, DCA, CDCA, and CA, in the colon inhibited TGR5 activation, which subsequently affected IEC regeneration. The Li05 treatment increased the concentration of LCA, DCA, CDCA, and CA and elevated the expressions of TGR5 and LGR5, suggesting that Li05 may activate the TGR5-LGR5 pathway by influencing bile acid metabolism to maintain IEC regeneration.

SCFAs are important energy sources for IECs and regulate the function of IECs through different mechanisms that regulate their proliferation and differentiation [49]. Studies demonstrated that SCFAs can activate host G protein-coupled receptors to protect the intestinal barrier, maintain metabolism homeostasis, and regulate the immune system, which facilitate liver regeneration and alleviate liver injury [50]. Our previous experiment indicated that Li05 achieved anti-inflammatory effects through increasing SCFA production [19]. Butyrate can decrease epithelial permeability and enhance tight junctions by increasing the expression of occludin, zonulin, and claudins [51], thereby inhibiting immune cell activation and pro-inflammatory cytokine production in liver injury [52]. Meanwhile, administration of appropriate concentrations of propionate and butyrate can promote MUC2 expression by regulating the promoter [53]. In this study, administration of Li05 increased propionic and butyric acid concentrations, which played a role in maintaining intestinal homeostasis and preventing further LPS-induced liver inflammation.

## 5. Conclusions

Overall, Li05 treatment significantly alleviated hepatic injury, cholestasis, hepatic inflammation, and fibrosis in the mouse model by activating the FXR-SHP and FXR-FGF15 pathways. Meanwhile, it protected the intestinal barrier and reduced bacterial translocation by increasing BA and SCFA concentrations and activating the TGR5-LGR5 pathway. As shown in our study, Li05 might be applied as a potential therapy to attenuate PSC.

## Data Availability

All the data in this study are presented in the paper and the Supplementary Materials. Additional data relevant to the article are available from the corresponding author upon request.

## Supplementary Materials

The following supporting information can be downloaded at: https://www.mdpi.com/article/10.3390/nu15234864/s1, Table S1: Primer sequences used for real-time PCR analysis and 16S rRNA. Figure S1: Immunohistochemical quantitative analysis.
