# Supplementary material for: Probiotic Pediococcus pentosaceus Li05 Improves Cholestasis through the FXR-SHP and FXR-FGF15 Pathways
Source: Nutrients. 2023 Nov 22;15(23):4864. doi: 10.3390/nu15234864 (PMC10708340; doi:10.3390/nu15234864)
Supplement: Supplementary file 1 [file nutrients-15-04864-s001.zip › Figure S1.pdf]

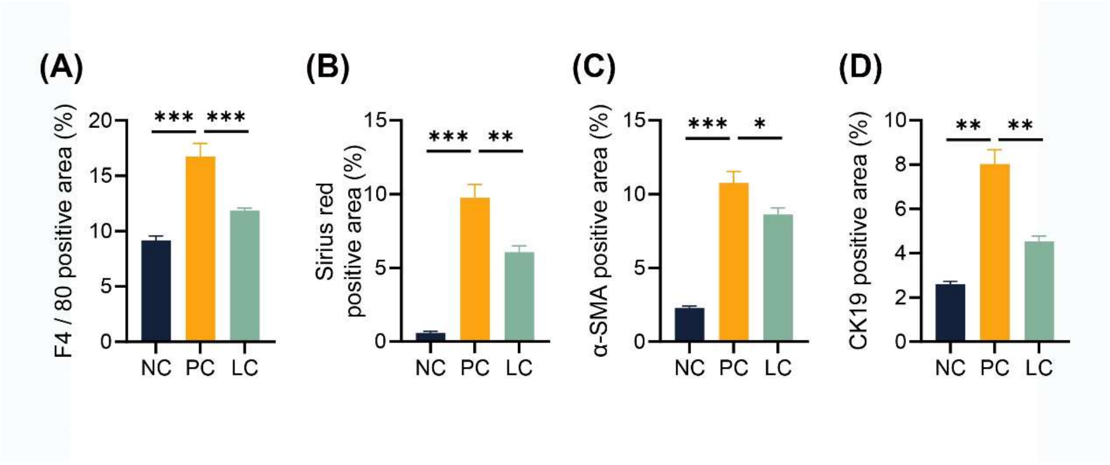

Figure S1. Immunohistochemical quantitative analysis. A-D) F4 / 80, Sirius red, α-SMA and CK19 positive area proportion. Data are presented as mean ± SEM, $*P < 0.05$, $**P < 0.01$ and $***P < 0.001$ according to the one-way ANOVA test.
