# Supplementary material for: Probiotic Pediococcus pentosaceus Li05 Improves Cholestasis through the FXR-SHP and FXR-FGF15 Pathways
Source: Nutrients. 2023 Nov 22;15(23):4864. doi: 10.3390/nu15234864 (PMC10708340; doi:10.3390/nu15234864)
Supplement: Supplementary file 1 [file nutrients-15-04864-s001.zip › Table S1.pdf]

Genes	Primer sense (5′-3′)	Primer antisense (5′-3′)
β-actin	AGTGTGACGTTGACATCCGT	GCAGCTCAGTAACAGTCCGC
TNF-α	CCCTCACACTCAGATCATCTTCT	GCTACGACGTGGGCTACAG
IL-1β	GAAATGCCACCTTTTGACAGTG	TGGATGCTCTCATCAGGACAG
IL-6	TCTATACCACTTCACAAGTCGGA	GAATTGCCATTGCACAACTCTTT
F4 / 80	TGACTCACCTTGTGGTCCTAA	TGACTCACCTTGTGGTCCTAA
Collagen 1a1	CTGGCGGTTCAGGTCCAAT	TTCCAGGCAATCCACGAGC
Collagen III	CTGGTCAGCCTGGAGATAAG	ACCAGGACTACCACGTTCAC
α-SMA	TAGTCCTTCCTACCCCAATTTCC	TTGGTCCTTAGCCACTCCTTC
TGFβ	CCACCTGCAAGACCATCGAC	CTGGCGAGCCTTAGTTTGGAC
CTGF	GGGCCTCTTCTGCGATTTC	ATCCAGGCAAGTGCATTGGTA
TIMP1	GCATCTCTGGCATCTGGCATC	GGTATAAGGTGGTCTCGTTGA
Cyp7a1	AACAACCTGCCAGTACTAGATAGC	GTGTAGAGTGAAGTCCTCCTTAGC
Cyp27a1	CCAGGCACAGGAGAGTACG	GGGCAAGTGCAGCACATAG
FXR	TGTGAGGGCTGCAAAGGTTT	ACATCCCCATCTCTCTGCAC
SHP	TCTGCAGGTCGTCCGACTAT	CAGGCAGTGGCTGTGAGAT
FGF15	GCCATCAAGGACGTCAGCA	CTTCCTCCGAGTAGCGAATCAG
ASBT	ACCACTTGCTCCACACTGCTT	CGTTCCTGAGTCAACCCACAT
NTCP	CAAACCTCAGAAGGACCAAACA	GTAGGAGGATTATTCCCGTTGTG
BSEP	TCTGACTCAGTGATTCTTCGCA	GTGTAGAGTGAAGTCCTCCTTAGC
Ostα	TGTTCCAGGTGCTTGTCATCC	CCACTGTTAGCCAAGATGGAGAA
Ostβ	GATGCGGCTCCTTGGAATTA	GGAGGAACATGCTTGTCATGAC
MDR1	ACACTTGGCCCCAAACATAGA	GTCAATGCTTGGCTCGTTATCA
MDR2	CGGCGACTTTGAACTAGGCA	CAGAGTATCGGAACAGTGTCAAC
MRP2	ACGTTTAGTTGGTATGACAGCAC	TGCTTCTTGGTCAATCCGTGT
V3-V4	ACTCCTACGGGAGGCAGCAG	GGACTACHVGGGTWTCTAAT
ZO-1	GCCGCTAAGAGCACAGCAA	GCCCTCCTTTTAACACATCAGA
Occludin	TGAAAGTCCACCTCCTTACAGA	CCGGATAAAAAGAGTACGCTGG
MUC2	AGGGCTCGGAACTCCAGAAA	CCAGGGAATCGGTAGACATCG
TGR5	TGCTTCTTCCTAAGCCTACTACT	CTGATGGTTCCGGCTCCATAG
LGR5	ACATTCCCAAGGGAGCGTTC	ATGTGGTTGGCATCTAGGCG

Table S1. Primer sequences used for RT-qPCR.
